# Asymptomatic aortic stenosis: An assessment of patients’ and of their general practitioners’ knowledge, after an indexed specialized assessment in community practice

**DOI:** 10.1371/journal.pone.0178932

**Published:** 2017-06-05

**Authors:** Raphaëlle-Ashley Guerbaii, Gabriel Fustier, Pierre-Vladimir Ennezat, Anne Ringle, Camille Trouillet, Pierre Graux, André Vincentelli, Christophe Tribouilloy, Sylvestre Maréchaux

**Affiliations:** 1 Department of cardiology, Centre Hospitalier Régional Universitaire Grenoble-Alpes, Grenoble, France; 2 Department of cardiology and heart valve center, GCS-Groupement des Hôpitaux de l’Institut Catholique de Lille (GHICL), Faculté de Médecine et de Maïeutique de Lille / Université Catholique de Lille, Lille, France; 3 INSERM U 1088 Université de Picardie, Amiens, France; 4 Department of clinical research, GCS-Groupement des Hôpitaux de l’Institut Catholique de Lille (GHICL), Faculté de Médicine et de Maïeutique de Lille / Université Catholique de Lille, Lille, France; 5 Department of cardiac surgery, Centre Hospitalier Régional Universitaire de Lille, Lille, France; 6 Cardiology B department, Centre Hospitalier Universitaire d’Amiens, Amiens, France; University of Milano, ITALY

## Abstract

**Background:**

Clinical and echocardiography follow-up is recommended in patients with aortic stenosis to detect symptom onset, thus a watchful waiting approach has to be safe and effective. For both AS patients and their general practitioners, evaluation of valvular heart disease (VHD) knowledge, after the indexed specialized assessment has never been measured.

**Aims:**

To evaluate the knowledge of clinical symptoms of aortic stenosis by both patients and their general practitioner.

**Methods:**

Sixty-four patients, with moderate to severe and initially asymptomatic AS (median AVA (interquartile range) 1.01(0.80–1.15) cm^2^) previously referred to a tertiary center and medically managed for at least 6 months after the index echocardiogram, and their primary care doctors were interviewed on the phone and asked to answer specific questions related to knowledge of aortic stenosis symptoms.

**Results:**

Fifty-six percent of patients quoted shortness of breath as one of the aortic stenosis symptoms, and only 16% knew the 3 aortic stenosis symptoms. Fifty percent of patients reported having received sufficient information regarding aortic stenosis; only 48% remembered receiving information regarding specific symptoms. Only 14% general practitioners quoted the 3 specific symptoms. According to the initial recommendation, only 41 patients (64%) benefitted from a 6-to-12 month clinical and echocardiography follow up.

**Conclusion:**

GPs are not sufficiently trained to safely manage AS patients in the community and to ensure adequate follow-up and monitoring. AS patients were not properly informed about their diagnosis and symptomatology. Hence, therapeutic education should be improved for patients with asymptomatic AS and continuous medical education on VHD should be reinforced, for GPs.

## Introduction

Over the last fifty years the epidemiology of valvular heart disease (VHD) has considerably changed in developed countries.[1] While a steady increase in life expectancy has been accompanied by a progressively increasing frequency of degenerative valve disease, rheumatic disease is now uncommon.[1] Aortic stenosis (AS) is the most frequent form of valvular disease in the industrialized countries and is relatively common in patients over 65 years old, with a 2% prevalence.[2] For patients with severe AS, symptoms such as dyspnea, syncope/dizziness and/or angina, or left ventricular ejection fraction (LVEF) <50% are strong indicators for aortic valve replacement (AVR).[3] Genuine asymptomatic AS patients should be conservatively managed until spontaneous symptoms occur, thus clinical and echocardiography follow-up every 6-to-12 months is recommended, to detect symptom onset. To ensure that this watchful waiting approach is safe and effective, primary care general practitioners (GPs) must be appropriately informed and trained in order to manage these patients accordingly. However, for both AS patients and their GP, evaluation of VHD knowledge after the indexed specialized assessment has never been measured.

## Materials and methods

The aim of this present investigation is to evaluate the knowledge of clinical symptoms of AS by 1) AS patients previously referred to a tertiary center and medically managed for at least 6 months after the index echocardiogram and 2) their primary care GP. Among all consecutive patients diagnosed with aortic stenosis, in the echocardiography laboratory of Saint Philibert Hospital (GHICL, Lille Catholic University, France) and included in a prospective registry[4], sixty-nine patients referred for exercise stress echocardiography of AS were retrospectively identified. Exclusion criteria were (*i*) left ventricular ejection fraction (LVEF) < 50%, (*ii*) more than mild-to-moderate concomitant aortic or mitral regurgitation.

Clinical evaluation was standardized and included assessment of cardiac and extra-cardiac comorbidities and current medication. The study was conducted in accordance with institutional policies, national legal requirements, and the revised Helsinki declaration and obtained ethical approval from the Comité Consultatif sur le traitement de l’information en matiére de recherche dans le domaine de la santé (CCTIRS).

Echocardiograms were performed on commercially available ultrasound machines (Philips IE 33, Epiq 7 and General Electrics Vivid E9) and stored on dedicated workstations (GE EchoPAC PC and Philips X celera) for offline analysis. Standard echocardiographic parameters were collected according to current EACVI/ASE guidelines for the practice of transthoracic echocardiography[5], by senior expert echocardiography and VHD cardiologists. Mean transaortic gradient and maximal aortic jet velocity were obtained using the non-imaging transducer and multiple views. Doppler Velocity Ratio and aortic valve area (AVA) were also calculated, taking account the pressure recovery phenomenon if the aorta at the tubular junction was less than 30 mm. Severe aortic stenosis was defined as an AVA < 1 cm^2^.

Phone calls were made to these patients and their GP at least 6 months after the initial evaluation, and were asked to answer the following questions:

1. Questions asked to patients:
  - Do you know the symptoms of your VHD? Please cite the symptoms you know (Expected answers: shortness of breath, dizziness /syncope, angina).
  - What would you do if you experience one the following symptoms: shortness of breath, syncope or angina. (Expected answers: a consultation with any of the following: GP, private cardiologist or specialized VHD cardiologist; or phone call to the emergency services).
  - Do you think that you understand your disease sufficiently (expected answer: yes or no)
  - Did you receive specific information about AS symptoms? (Expected answers: yes or no)
2. Questions asked to GPs:
  - Do you know AS symptoms? Please cite the symptoms you know (Expected answers: shortness of breath, dizziness/syncope, angina)
  - To which physician would you refer your patient to, if AS symptoms occur? (Expected answers: cardiologist, cardiac surgeon, heart valve disease specialized cardiologist)

Oral informed consent was obtained from all study participants.

Continuous variables are expressed as mean ± standard deviation or median (25^th^– 75^th^ percentile-Inter Quartile Range (IQR)) in case of skewness. Categorical variables are expressed as absolute numbers and percentages. Categorical variables were compared between patients with and without severe AS using the Pearson’s Chi square test or the Fisher exact test as appropriate. A 2-tailed P-value ≤ 0.05 was considered for statistical significance. Statistical analyses were performed using SPSS 21.0 (IBM Corp. Armonk, NY).

## Results

**Fig 1:**
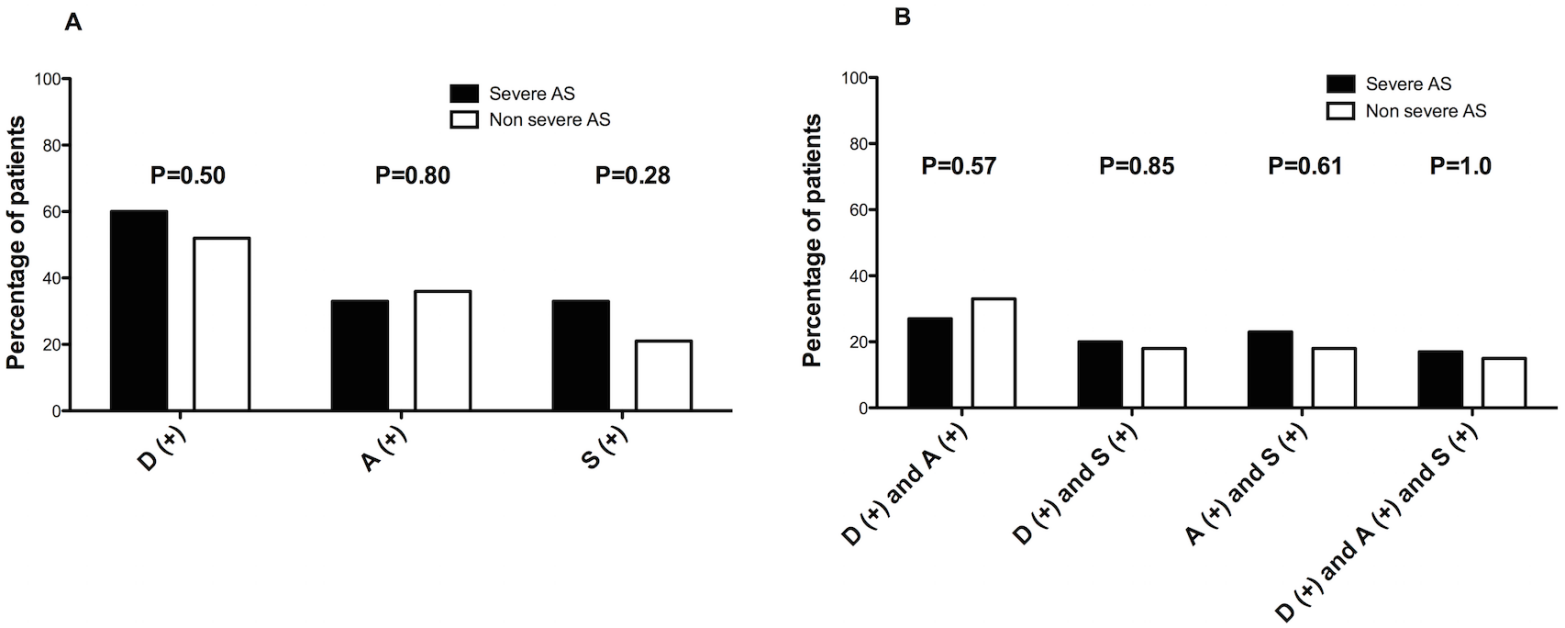
(A) Frequency of the knowledge by patients of Dyspnea (D(+)), Angina (A(+)) or Diziness/Syncope (S(+)) as a symptom of aortic stenosis (AS) according to the severity of AS (severe versus non severe). (B): Frequency of the knowledge by patients of 2 symptoms (D(+) and A(+), D(+) and S(+), A(+) and S(+) and of 3 symptoms of AS according to the severity of AS (severe versus non severe).

Two patients were lost to follow-up, two patients refused to participate, 1 patient was not able to answer the questions and were subsequently excluded, hence resulting in a study population of 64 patients. Clinical and biological characteristics of the study population are detailed in Table 1. Severe comorbidities were rare in this population of asymptomatic AS patients referred for exercise stress testing. Mean patient age was 71 years old and 50% of patients were female. As shown in Table 2, LVEF was normal (64±7%) in all patients. Mean transaortic gradient was 36±14mmHg and maximal transaortic jet velocity was 3.8±0.7 m/s. Thirty patients (47%) had severe AS (AVA < 1cm^2^) and median AVA (IQR) was 1.01 (0.80–1.15) cm^2^. No deaths were recorded during follow-up. Thirty-six patients (56%) quoted shortness of breath as an AS symptom, angina was cited in 22 (34%) and dizziness/syncope in 17 (27%). Forty-four patients (69%) knew 2 AS symptoms (19 patients quoted dyspnea + angina, 12 quoted dyspnea + dizziness/syncope and 13 quoted angina + dizziness/syncope) and only 10 patients (16%) knew the 3 AS symptoms. The relationship between awareness of symptom knowledge and the severity of AS (severe versus non severe) is depicted in Fig 1A and 1B. Patients with either severe or moderate AS at baseline had similar AS symptom knowledge. In the event of spontaneous symptoms, 26 patients (41%) contacted their private cardiologist, 21 patients (33%) contacted their GP, 15 (23%) called the emergency services number and 4 patients (6%) contacted the tertiary heart valve center. Thirty-two patients (50%) reported being sufficiently informed of the development of their valvular disease. However, only 31 patients (48%) remembered having received information regarding specific AS symptoms. One patient was found symptomatic with obvious dyspnea at time of the phone call without having had a cardiology evaluation in the past 2 years. This patient was subsequently referred for AVR after preoperative evaluation.

**Table 1:** Demographic and clinical characteristics of the study patients.

**Clinical data**	
Age (years)	71 ± 11
Female (n,%)	32 (50%)
Body Mass Index (kg/m^2^)	27 ± 5
NYHA functional class I II	54 (84%) 10 (16%)
Diabetes mellitus (n,%)	19 (30%)
Dyslipidaemia (n,%)	39 (61%)
Smokers (n,%)	6 (9%)
Hypertension (n,%)	53 (83%)
History of coronary artery disease (n,%)	18 (28%)
Atrial fibrillation (n,%)	6 (9%)
Chronic renal failure (n,%)	2 (3%)
Chronic pulmonary disease	10 (16%)
Previous cardiac surgery (n,%)	5 (8%)
**Biological data**	
Brain natriuretic peptide (pg/mL)	62 [39–131]
Urea (mg/dL)	0.36 (0.27–0.40)
Creatinin (mg/dL)	9.3 (7.7–10.5)
Hemoglobin (g/dL)	12.9 (12.0–13.8)

**Table 2:** Echocardiography parameters.

Systolic blood pressure (mmHg)	140 ± 17
Diastolic blood pressure (mmHg)	74 ± 10
Heart rate (beats per minute)	74 ± 15
LV end-diastolic diameter (mm)	45 ± 6
LV end-systolic diameter (mm)	27 ± 6
IVS diastolic thickness (mm)	12 ± 2
LV posterior wall diastolic thickness (mm)	12 ± 2
LV mass index (g/m^2^)	109 ± 30
Relative wall thickness	0.56 ± 0.14
LV end-diastolic volume (mL)	115 ± 38
LV end-systolic volume (mL)	42 ± 18
LV ejection fraction (%)	64 ± 7
Doppler stroke volume index (mL/m^2^)	45 ± 9
LV outflow tract diameter (mm)	22 ± 2
Aortic sinus diameter (mm)	33 ± 4
Sino-tubular junction diameter (mm)	29 ± 4
Tubular aorta diameter (mm)	35 ± 5
E/A ratio	0.84 ± 0.38
Mitral deceleration time (ms)	235 ± 68
Left atrium volume index (mL/m^2^)	39 ± 14
Pulmonary artery systolic pressure (mmHg)	32 ± 7
Mean transaortic gradient (mmHg)	36 ± 14
Aortic maximal jet velocity (m/s)	3.8 ± 0.7
Aortic valve area (cm^2^)	1.01 ± 0.27
Doppler Velocity Ratio	0.26 ± 0.07

LV: left ventricular, IVS: interventricular septum

The 69 patients initially screened for the study had 67 GPs. Among GPs, 58 (87%) GPs out of the 67 GPs selected, agreed to respond to the present investigation. Shortness of breath was quoted as an AS symptom by 52 GPs (90%), angina was quoted by 26 (45%) and dizziness/syncope was only quoted by 15 (26%). Surprisingly, three GPs (5%) did not quote any symptoms, twenty-two GPs (38%) knew 2 symptoms of AS (16 dyspnea + angina, 4 dyspnea + dizziness/syncope, 2 angina + dizziness/syncope) and only 8 GPs (14%) quoted the 3 AS symptoms. Sixty-one GPs (97%) addressed symptomatic patients to their private cardiologist and 2 GPs (3%) to the heart valve disease specialized cardiologist. According to the initial recommendation, only 41 patients (64%) benefitted from a 6-to-12 month clinical and echocardiography follow up.

## Discussion

The initial management of asymptomatic patients with VHD is usually conservative, thus, meticulous follow-up is important. Furthermore, the application of accepted guidelines requires specialist experience especially in determining whether a patient is genuinely asymptomatic.[6] General cardiologists or GPs, who may be less skilled than a VHD specialist in making a diagnosis, and formulating a treatment plan, care for most patients suffering from VHD. Furthermore, it is likely that advances in practice are more slowly assimilated by a GP than by a cardiologist who undertakes specialist continuing medical education.

This study demonstrates weaknesses associated with the management of asymptomatic AS patients in the community. According to current guidelines[3, 7], asymptomatic AS patients should be closely monitored, until spontaneous symptoms occur (ie watchful waiting strategy). Prophylactic AVR is usually performed in asymptomatic AS patients with poor short-term outcome including very severe AS and an abnormal exercise test.[3, 7–9] Surprisingly, in the present cohort of asymptomatic patients with moderate to severe AS and conservatively managed in the community (during an initial period of at least 6 months), specific directives from VHD specialists regarding follow up were not widely received. Only, 48% of patients remembered having received information about AS symptoms during the initial evaluation, suggesting that these patients are not given oral or written material to help identify onset of symptoms. In addition, 86% of GPs did not monitor the 3 specific AS symptoms during routine check-up, which were quoted by only 14% of GPs. Although no patients died during follow up, 1 out of 64 patients had overt AS symptoms at the time of the investigation phone call. Recently, the European Society of Cardiology has emphasized the importance of the development of heart valve clinics (HVC) [10], in order to specifically monitor and detect onset of symptomatic AS and other VHDs. Zilberszac et al have recently demonstrated that structured HVC programs enable timely management of symptoms at an earlier and less severe stage and thus an optimized timing of surgery.[11] The aim of these clinics is to deliver standardized care, using patient tailored protocols, to ensure coordination of care between GPs and cardiologists, thereby improving clinical management of VHD patients.[10] Therapeutic education of patients including symptom recognition and reporting would be accomplished. Training and implementing specialized clinical nurses in HVCs would enable in-depth therapeutic education, such as recognizing both symptom onset and severity, but also phone call monitoring of VHD patients between follow-up appointments, to avoid rapid deterioration in case of symptom onset. In addition to providing support and guidance to stable AS patients, this would also be a cost-effective strategy as patients are kept in the community and out of hospital.

The mechanisms leading to the development of aortic valve stenosis lesions were traditionally believed to be degenerative, induced by time-dependent wear and tear of the leaflets with passive accumulation of calcium in the setting of sclerosis.[12] However, atherosclerotic risk factors have been associated with aortic sclerosis[13, 14], and histological analysis has revealed atherosclerotic- like lesions in aortic leaflets, characterized by accumulation of macrophages, T lymphocytes, oxidized low-density lipoproteins (LDL), and extracellular lipids at the aortic side of the leaflets.[15–17] To this effect, GPs may play an important role in the prevention and management of atherosclerotic risk factors in patients with AS in order to limit AS progression. Hence, specialized cardiologists in HVC may help to educate GPs in the management of AS in terms of aggressive risk factor management including cholesterol or blood pressure.

Despite clear advantages, Bhattacharyy et al, reported significant differences as 60% of HVCs were sited in tertiary centers, compared with only 11% in district hospitals in the United Kingdom for instance, which suggests that inequalities may occur.[18] It was also established that one third of these clinics were led by specialist nurses and sonographers, which also underlines the potential for training health professionals in order for HVCs to develop in France. Preliminary evidence demonstrates that this is a safe and cost-effective strategy, when supervision from a specialized cardiologist is implemented.[18] However, organizational problems with HVCs may occur, as uncomplicated patients may be discharged back to the community and subsequently lost to follow-up. These findings have been confirmed in a small survey, which confirms that efficient community follow-up does not always occur, and therefore patients do benefit from specialist follow-up.[19] Additionally, the present report shows that only 41% of patients contacted their cardiologist in the event of symptom development. Furthermore, GPs also have an important role in the detection of symptoms in patients without self-reporting symptoms despite being truly symptomatic.

### Study limitations

Due to the small sample size of study participants, our findings may not be representative of larger communities. The inclusion of patients with moderate AS may represent a limitation. However, the outcome of patients with moderate AS is not benign, and patients with moderate AS can rapidly progress to severe symptomatic AS requiring surgical management. Thus, reinforcing the importance of therapeutic education and close follow up in patients with moderate AS is essential.[20] It is of importance to note that GPs roles do differ between countries, hence the results of this French study may not be generalizable.

## Conclusion

This study suggests that GPs are not sufficiently trained to safely manage AS patients in the community and to ensure adequate follow-up and monitoring. In addition to this, AS patients were not properly informed about their diagnosis and symptomatology, thus suggesting that these patients are not sufficiently involved in their disease and are not able to detect clinical deterioration. Hence, therapeutic education should be improved in patients with asymptomatic AS and GPs continuous medical education on VHD should be reinforced. The development of HVCs may help to ensure adequate care and monitoring of patients with VHD, and a better interplay between specialized centres and general practice in the community.

## Supporting information

S1 Table Data set. Data set of the study.
(XLSX)
